# Hospital Meetings, &c.

**Published:** 1900-11-10

**Authors:** 


### The Institutional Workshop.

### HOSPITAL MEETINGS, &c.

### HOSPITAL SATURDAY FUND.

The Annual Public Meeting of the Hospital Saturday Fund was held on the 3rd inst. at the Mansion House, the Lord Mayor (Sir Alfred Newton) opening the proceedings and being succeeded in the chair by Mr. R. B. D. Acland when obliged to leave to fulfil another engagement. The meeting was well attended, the great hall being full. The twenty-sixth annual report of the Council states that the year 1899 was one of steady progress. Notwithstanding the fact that it was only the second year since the abolition of the Annual Street Collection, and that in the last quarter, the many claims of the sufferers by the war in South Africa might have accounted for some considerable diversion of income, the total amount received was £20,013, being £951 in excess of the income for 1898, and only £125 less than the amount collected in 1897, the last year of the Street Collection. The Local Committees are recovering from the change of methods necessitated by the discontinuance of the Annual Street Collection, and some have collected as much as they did in any previous year. The total sum raised through the influence of the Local Committees in 1899 was £1,473, as against £1,117 in 1898. The revised regulations for Local Committees, as approved by the Board of Delegates on June 10th, 1899, are calculated, when faithfully carried out, to secure the confidence of the public. The total sum distributed for 1899 was £17,013, leaving a balance at the bank of £1,013. Thirty-six General Hospitals received £6,547; sixty Special Hospitals received £4,989; and seventy-eight miscellaneous institutions received £5,477. The important principle of self-help is inculcated by the Association with increasing success. This is done by means of the part-pay system for maintaining convalescents at homes, and for providing patients with surgical appliances. Whilst 908 patients were sent with free letters to convalescent homes during the year, no fewer than 430 were sent on payment of half the cost of maintenance. Similar success has attended the practice of requiring patients to pay in easy instalments half the cost of surgical appliances. In the case of artificial teeth, three-quarters of the cost are demanded. In the course of the year appliances to the number of 3,121 were supplied, the recipients paying £1,409 and the Fund £1,020 towards the cost. In their last Report the Council announced that it had been decided to appoint dentists in various parts of London to meet the demand for dental treatment and to prevent the loss of time in travelling and awaiting treatment at hospitals, but the surgical appliance committee has been obliged to make more stringent regulations with regard to the issue of letters of recommendation. The educational work of the Fund has progressed satisfactorily. Classes in first aid were held in the first and last quarters of the year. The two divisions of the St. John Ambulance Brigade connected with the Fund have rendered public service on many occasions. Sixty-seven ambulance boxes were in use in as many workshops. In addition to being of much practical use, these boxes were the means of disseminating knowledge in first aid. The arrangements with the invalid transport corps of the St. John Ambulance Association, for the removal of patients to or from hospitals in horse ambulance carriages with attendants, worked most satisfactorily, and only require to be better known to be more extensively used. The expenses of management last year amounted to £2,191, which is at the rate of rather less than 11 per cent. of the gross receipts. This percentage is lower than that of any previous year. The number of letters received by post during the year was 26,258, plus an estimate of 19,750 for letters delivered by hand. The total number of meetings of the Board and Standing Committees was 231. The number of letters of recommendation issued was 25,797. The number of receipts issued from the Head Office in the course of the year was 12,100. During the year the sum of about £50 per week is received in bronze coins. This means that over five and a half tons of bronze coins have to be recounted, packed, and finally exchanged before being paid into the account at the bank. The Council are concerned with regard to the present position of the Premises Fund. The statement of accounts shows that the debt stands now at £4,134. The clearing off of this debt will be a perpetual endowment of at least £200 per annum. The success of the Fund during the last three years is in no little measure a result of the improved accommodation provided by these freehold premises, and the Council earnestly hope that well-wishers of the Fund will help in some substantial way to clear off this debt as soon as possible. The relations of the Fund with the other collecting agencies and the participating institutions have been marred with no unpleasantness during the year. The Council of the Prince's Fund has shown the kindest consideration for the work of the Hospital Saturday Fund, and, whenever possible, the heartiest co-operation for the good of the London hospitals. Sir Savile Crossley, Bart., was unanimouely elected Chairman of the Fund on February 17th, 1900. Being already connected with the Prince of Wales's and Sunday Funds, as Chairman of the Saturday Fund he may be enabled to forward the policy of not only preventing any clashing between the three Funds, but of drawing them, if possible, closer together. The cordial thanks of the Council are accorded to the Honorary Auditors, Messrs. W. H. Pannell and Co., and to the London Press and to the subscribers and collectors for their continued support during the year, and the hope is expressed that the same earnest endeavour will be made during 1900 as was made last year for the London hospitals, which will have to provide not only for the afflicted who are always with us, but also in a measure for the sick and wounded soldiers, sailors, and volunteers, who will unfortunately require the best treatment that the London hospitals and the convalescent homes connected therewith can afford.

The Lord Mayor in a brief address expressed satisfaction that the sympathies of the working classes should be enlisted in favour of the hospitals, which it was a matter of justice and fairness that they should support. He asked the meeting to excuse his departure as he had to attend a prize distribution at the Guildhall School of Music, and requested Mr. Acland to take his place.

After the Secretary had notified the receipt of letters of regret from the Bishops of London and of Islington, Canon Fleming, the Revs. Samuel Barnett, F. B. Meyer, and Thomas Spurgeon, and Sir Mancherjee Bhownaggree, M.P.,

Mr. R. D. B. Ackland made his customary annual statement as to the position of the Fund. He did so, he said, though as they knew he was not now Chairman of the Fund. Their present Chairman, Sir Savile Crossley, was engaged in fighting his country's battles in South Africa, and he was sure they all wished him a safe return. Although up to October 27th this year they had only collected £12,697, as compared with £12,976 last year, yet he hoped that the falling-off was only apparent. He attributed it to the effects of the war, and the many calls in connection with it, and he thought the fact that many firms, in addition to their hospital collections, contributed with regularity to the various war funds, spoke volumes for the innate charity of the British workman. And they had some gleams of light to comfort them: for instance the Projectile Company—who he supposed had been making so much they could afford to do a little more—with £12, and Marshall and Snellgrove with £69, showed increases on last year's amounts. The Arsenal, at Woolwich, also showed a considerable increase. He should also like to call attention to two new developments taken at the initiative of the employers themselves, and well worthy of imitation. At the Millwall Docks application had been made to the firms who used the docks, and several guineas had resulted; and Messrs. Garrould had had a collecting box on the occasion of the C.I.V. procession which had resulted in the substantial sum of £13 being obtained. Another hopeful sign was that during the present week over £500 had come in, as against £301 in the corresponding week of last year; and altogether he did not think they were going to be backward in the end. Referring to the work of the local committees under the new conditions, he said that now the street collections had been given up—greatly to the credit of the Fund and of London generally—it involved the change from itinerant beggars to steady, hardworking collectors. He appealed to all tradesmen to do what they could to help by taking boxes and otherwise. And everyone could help by having a box at home, and so give their friends an opportunity of contributing if they wished to. During the year they had had to deplore the loss of two men who had done infinite service to the organisation—Mr. Yerbury and Mr. Dickinson. To Mr. Yerbury they owed their present scheme of award. Mr. Dickinson was one of the founders of the Fund, and the idea of it, if not conceived, was at all events fostered by him, and he lived to see its success. His tragic death was due to the strain of devoting his leisure to helping those about him. He doubted if there was ever a man who in so humble a position was able with his own hands and by his own efforts to produce so great effects. He had laid down his life as a true martyr to the cause of others.

Sir Henry Burdett moved the following resolution:—

> "That this meeting approves of the principle of soliciting small weekly contributions in aid of the Metropolitan medical charities from those engaged in the workshops, warehouses, and places of business in the Metropolis."

He said he had in the last 20 years attended many meetings for philanthropic purposes, and as one who was largely interested in hospital matters he found it a matter of experience that there was an encouragement about Hospital Saturday Fund meetings often lacking about others, due to the large attendance always to be found. They afforded an example of the vitalising influence of personal and individual interest. It was a great and important fact, and none who loved London could fail to recognise that London was on its trial, and that the safety of London depended on the individual. He took it that no one could have looked with satisfaction on what occurred on the previous Monday when they welcomed back the City Imperial Volunteers. The excesses and the license on that occasion were matters for deep and real regret, and all decent people must have felt sick at heart that there should have been such an exhibition of lawlessness in our city. It was essential that self-restraint and self-respect should be cultivated. He hoped also that the powerful appeal from Lord Roberts to all to abstain from tempting the returning soldiers to drink would be heeded, and that we should welcome them as sane men and women, conscious of our nationality and proud of it and its solid and sober characteristics. (Loud applause.) They had just passed through the first municipal election under the new law, which was an attempt on the part of the Legislature to get individual citizens to take their part in local government in such a way as not to be lost in the great mass of the population. He hoped as one result that there would be an exhibition of generosity and unselfishness and courage which would have an influence for good on the whole of the population. He took it that there were two vital questions which concerned all who earned their living with the sweat of their brows—provision for old age and provision for sickness. He thought it might legitimately be pointed out to the Government that the time had arrived when the Old-age Pension question should be closed by putting within the means of all who labour the power to provide for the time when they were no longer able to do so. There could be no possible doubt that the question could be solved without unduly trenching on the public exchequer. But the scheme must be on a thrift basis; the people only required the necessary facilities to supplement the efforts they themselves were prepared to make. As to the question of provision for sickness, he considered the principle underlying the Saturday Fund was a thrift principle and therefore sound. He wished to emphasise his approval of the weekly contributions because everybody should, in the day of health, take such steps as were within their means to be sure of having relief when the day of sickness came. Wages were paid weekly and they had then the opportunity of putting aside for that day. He took the fact that it had survived so much criticism as evidence that they had in the weekly contribution system a sound and lasting principle. He should like the Hospital Saturday Fund to consider the idea of using as a subscription book the stamp album published for the purpose. It enabled the subscription to be recorded in an unquestionable manner, and the contributor was thus able to carry about with him indisputable evidence of the fact that he had contributed toward the support of the hospitals. He was in a position to offer the Council a large number of the books and of stamps, provided they were issued to contributors, and the face value of the stamps represented the amount contributed. On the subject of hospital abuse he knew it was declared to be continually extending. The population of London had increased by 800,000 in ten years, and the hospital population by about 400,000 in the same time. That seemed to show that one out of every two of the addition resorted to hospitals when ill. But he did not think that was quite the fact. Travelling facilities were now so great that a large number of cases were sent from the home counties into the London hospitals. From a day census of the hospitals it appeared that some 15 per cent. of the occupants of the beds came from the home counties, and among out-patients the proportion was rather larger. And it must also be remembered that at the present time there were no means available for people in relatively humble circumstances whereby they could get hospital accommodation provided at a cost within their means. It was high time that hospital authorities looked in the face the question of hospital abuse. What was required was that they should extend their system, and build on the sites at their disposal wards in which people could be received, and pay for their treatment. It was not fair to talk of abuse when the only alternative to very many was to incur very great risks by remaining outside and without the advantages obtainable within the hospital walls. He thought the time had come to learn from hospitals abroad, which were based on the plan that everybody paid. There were four grades. For the lowest the Poor Law authorities or the municipality paid, for the others the patient or his friends in varying degree up to the highest scale of 4 to 5 guineas a week. Eleemosynary medical attendance should be altogether abolished, and all medical men be paid. There could be a separate entrance to the pay-ward, so that the patient's own medical attendant could enter and leave independently of the general hospital, and it would be very easy to organise a system whereby patients could be attended by their own medical man and a proportion of the payment to the hospital set aside for their payment. He had been approached with reference to a proposal that it would cut at the root of the hospital abuse if we had a great insurance company which, for small regular payments, would be prepared to provide payment for sickness, with an understaking in the policy to pay for treatment when in the pay-ward of a hospital. He threw out this suggestion as indicating the direction in which public opinion was trending. They had only to consider that great institution, the Prudential Assurance Company, with its almost military despotism, to realise that there need be no insuperable administrative difficulty. After referring to the vitalising influence of individualism in the board rooms of hospitals and the difficulty of finding people willing and capable of taking seats on hospital committees, Sir Henry concluded with a warm eulogy of the Chairman of the Council, Sir Savile Crossley, who, he said, went out to South Africa entirely from a high sense of duty.

Dr. Lister seconded the motion, which was supported by Mr. Bousfield, who spoke of the Fund as one of the great friendly societies which were the glory of our English town life, and said no system could be permanently successful under which the medical profession—than whom there was no more charitable body of men in the country—did not get due payment and due honour for work done.

The motion was agreed to.

The second Resolution—

> That this Meeting cordially approves of the efforts of the Hospital Saturday Fund to increase the interest of the working classes in the work of the Metropolitan Medical Institutions—

was moved by Mr. George Howell, who said the object of the Fund was to get the working classes to help themselves and help each other—one of the forms of self-help which had given character to this country. They wanted, as regarded medical treatment, that the working classes should provide against the day of calamity. He emphasised the need for regular, weekly, and cheerful contributions.

Dr. Tom Taylor seconded, and gave particulars of the ambulance work done. The resolution was supported by the Rev. W. J. Ferrar and carried *nem. con.*

A vote of thanks to the Lord Mayor for the use of the Mansion House, moved by Mr. Hamilton-Hoare, and one to the Chairman, by Sir Henry Burdett, brought the proceedings to a close.

### THE EVELINA HOSPITAL FOR SICK CHILDREN.

The Hon. Conrad Dillon presided at the half-yearly Court of Governors of the Evelina Hospital for Sick Children, Southwark, on Wednesday, October 31st. The statement of accounts, he said, showed a balance of a few pence over £1,000 in hand to carry on for the next two months. The work of the hospital was going on quite satisfactorily, and the only undertaking calling for mention was the putting up of an efficient apparatus for disinfecting the children's bedding in case of necessity, and the replacing of the old laundry which had done duty for so many years by a steam laundry, by which the work would not only be better and more economically done, but the necessity avoided of having in women to do it—themselves a possible source of infection. Before putting in the disinfecting apparatus one of their friends had visited one of the large infectious hospitals with a view to learning whatever was best to be done in the matter. The greatest trouble in a children's hospital was always in the risk of wards having to be closed because of the unsuspected introduction of a case of diphtheria or fever. They would now be able to take all necessary steps properly and adequately. They had had a visit from two gentlemen representing the Prince of Wales's Fund; but though they had expressed themselves as entirely satisfied with everything, yet because they had spent £200 less than their income they received no grant. That, he thought, was rather hard lines.

There was no business to transact, and a vote of thanks to the Chairman closed the proceedings.

